# Know Your Epidemic, Strengthen Your Response: Developing a New HIV Surveillance Architecture to Guide HIV Resource Allocation and Target Decisions

**DOI:** 10.2196/publichealth.9386

**Published:** 2018-02-14

**Authors:** Brian Rice, Travis Sanchez, Stefan Baral, Paul Mee, Keith Sabin, Jesus M Garcia-Calleja, James Hargreaves

**Affiliations:** ^1^ Department of Social and Environmental Health Research Faculty of Public Health and Policy London School of Hygiene and Tropical Medicine London United Kingdom; ^2^ Department of Epidemiology Rollins School of Public Health Emory University Atlanta, GA United States; ^3^ Center for Public Health and Human Rights Department of Epidemiology Johns Hopkins School of Public Health Baltimore, MD United States; ^4^ Strategic Information and Evaluation Joint United Nations Programme on HIV/AIDS Geneva Switzerland; ^5^ HIV/AIDS Department World Health Organization Geneva Switzerland

**Keywords:** HIV, data, systems, surveillance, testing, treatment, prevention, monitoring, key populations

## Abstract

To guide HIV prevention and treatment activities up to 2020, we need to generate and make better use of high quality HIV surveillance data. To highlight our surveillance needs, a special collection of papers in *JMIR Public Health and Surveillance* has been released under the title “Improving Global and National Responses to the HIV Epidemic Through High Quality HIV Surveillance Data.” We provide a summary of these papers and highlight methods for developing a new HIV surveillance architecture.

In 2014, targets were set to diagnose 90% of all people living with HIV, provide antiretroviral therapy for 90% of those diagnosed, and achieve viral suppression among 90% of those treated by 2020 [1]. In 2016, a United Nations Political Declaration called on countries to achieve 500,000 fewer people newly infected with HIV, 500,000 fewer people dying from AIDS-related causes, and the elimination of HIV-related discrimination by 2020 [2].

To guide HIV prevention and treatment activities up to 2020 and beyond will necessarily require developing a new surveillance architecture that better delivers and leverages high quality HIV surveillance data. We must plan for more sustainable, country-led, action-oriented HIV surveillance platforms that can serve both local decision making and global reporting and modelling needs. In May 2015 the World Health Organization (WHO) and the Joint United Nations Programme on HIV/AIDS held their third global consultation meeting on HIV surveillance [3]. Over four days, discussions focused on recognizing priority gaps in current surveillance systems, identifying the surveillance data needed to monitor achievement of long-term goals such as the 90-90-90 indicators, and consolidating a global surveillance agenda to guide global and national programs. To reflect these discussions, we have released a special collection of papers in *JMIR Public Health and Surveillance* entitled, “Improving global and national responses to the HIV epidemic through high quality HIV surveillance data.” This special collection includes papers that present creative methods for developing, collecting, and using HIV surveillance data to guide HIV resource allocation and targeting decisions.

The galvanizing consensus around the 90-90-90 targets highlights our need to more accurately track the reach of testing and treatment programs. This will be best achieved by drawing on data generated through the delivery of these services rather than self-reports in surveys or the screening of non-HIV test blood specimens. A number of the papers in the special collection explore how existing information from HIV testing and treatment services can be valuable surveillance data, particularly for monitoring achievement of HIV care goals. Dee et al assessed how the move away from unlinked anonymous testing among pregnant women attending select antenatal clinics to surveillance based on routine HIV testing in these facilities may enhance our surveillance efforts [4]. The authors concluded that this shift represents a substantial achievement in building strong routine data systems to support HIV service delivery, program monitoring, and strategic information.

To ensure broader national surveillance and monitoring and evaluation (M&E) systems do not go the same way as antenatal sentinel surveillance, they must evolve. Rice et al and Bozicevic et al considered how best to strengthen existing systems [5,6]. Focusing on the use of routine HIV data collected in sub-Saharan Africa through service delivery platforms, Rice et al presented four priorities for action to drive more effective and efficient clinical management and prevention programing [5]. Bozicevic et al conducted a needs assessment of HIV strategic information systems in non-European Union countries in the WHO European region [6]. The authors identified a number of areas for capacity building to ensure these systems meet future needs, including improving their ability to disaggregate data by demographical and epidemiological factors.

A majority of national surveillance and M&E systems are limited in how information can be presented since they rely on the collection of aggregate data. To overcome the limitations of aggregate data, we need to develop comprehensive strategic HIV information systems that leverage individual-level data collected at HIV diagnosis and over time. Case-based surveillance (CBS) is such a system. Currently, no such system exists in sub-Saharan Africa, where the burden of disease is greatest. To identify systems that may be utilized in the development of CBS, Harklerode et al conducted situational assessments in Tanzania, South Africa, and Kenya [7]. To promote the collection and use of individual-level data through CBS, the authors made a number of recommendations based on their observations—recommendations that have informed recent global guidelines on person-centered HIV patient monitoring and case surveillance [7,8]. In their review of global surveillance priorities, Low-Beer et al outlined the fifteen steps required to develop a person-centered surveillance approach, five of these relate to CBS [9]. The remaining ten directions are categorized under improving patient monitoring and scaling up unique identifiers.

Commenting on the importance of surveillance data in program implementation, Low-Beer et al highlighted the need for integrating HIV activities with those related to sexually transmitted infections, hepatitis, and health [9]. In response to this, Hutin et al identified similarities and differences between the hepatitis and HIV response [10]. Concluding that integration or linkage is possible, the authors presented an approach to align the two streams of strategic information. Linkage across health sectors is something Nguyen et al call for in their analyses of a community-based retrospective cohort study among pregnant women diagnosed with HIV infection in Vietnam [11]. To bridge serious gaps in efforts to eliminate mother-to-child transmission, the authors endorsed data linkage across HIV and maternal and child health programs.

Despite understandable skepticism with regard behavioral surveillance, which has been undermined by social desirability biases and poor data quality, it will be critical to improve our capacity to track risk if we are to accurately predict epidemic pathways and reinvigorate HIV prevention efforts. To substantially reduce levels of HIV transmission, it is essential we improve strategic information among key populations, including better leverage of data on the size and location of populations most at risk. A number of the papers in the special collection focus on methods to improve surveillance activities with key populations at risk of HIV infection.

Reviewing the adequacy and relevance of current surveillance methods in key populations, Weir et al proffer a number of strategies to improve strategic information and estimates [12]. Among these is a recommendation to assess selection bias and subgroup representation. Analyzing data collected among female sex workers in Zimbabwe, Fearon et al investigated sample size effects on population size estimates using multiplier methods and respondent driven sampling [13]. A high variance in estimates was reported. Complementary to this work, Rao et al presented an analysis of eight studies on female sex workers and men who have sex with men, where participants were recruited using respondent-driven or venue-based snowball sampling in Swaziland and Cameroon [14]. The authors demonstrated how different sampling methodologies generate samples with a varying composition of HIV prevention needs and program exposure. In both of these analytic research papers, recommendations for reducing bias and improving population size estimates were presented.

To promote comprehensive HIV and general health programing, it is essential to include adolescents who sell sex, engage in same-sex relationships, and/or inject drugs. It is also important to integrate validated stigma scales to characterize key populations. These were the principal recommendation of Johnston et al [15] and Stahlman et al [16]. Arguing that adolescent groups disproportionately affected by HIV are largely ignored, Johnston et al put forward a number of suggestions for including these groups in future surveillance activities in a manner that is both ethical and effective [15]. With the intention of improving HIV-related outcomes, Stahlman et al outlined a vision for integrating validated stigma scales in key population epidemiologic, surveillance, and intervention studies [16].

As testing and treatment have expanded, so has the amount of data on HIV infections generated through service delivery. These data now dwarf those collected through bespoke research or surveillance activities. The rapid expansion of networked infrastructures provides an opportunity to develop a new surveillance architecture to harness these data at scale. The collection and secure storage of routine individual level data, linked within and across systems, will deliver more accurate, disaggregated and sustainable measurements of HIV incidence, HIV transmission risk, and HIV acquisition risk at a level and timeliness that can support resource allocation decisions and targeted action that will accelerate the reduction of HIV incidence.

## Abbreviations

WHO: World Health Organization
M&E: monitoring and evaluation
CBS: case-based surveillance
